# Suprasellar Melanocytoma with Leptomeningeal Seeding: An Aggressive Clinical Course for a Histologically Benign Tumor

**DOI:** 10.1155/2021/7306432

**Published:** 2021-10-11

**Authors:** Imen Maaloul, Marwa Moussaoui, Ameni Salah, Wiem Feki, Hela Fourati, Nadia Charfi, Zeineb Mnif

**Affiliations:** ^1^Radiology Department, Hedi Chaker Hospital, Sfax, Tunisia; ^2^Faculty of Medicine of Sfax, Tunisia; ^3^Faculty of Medicine of Monastir, Tunisia; ^4^Endocrinology Department, Hedi Chaker Hospital, Sfax, Tunisia

## Abstract

**Introduction:**

Meningeal melanocytoma (MM) is a very rare neuroectodermal neoplasm arising from the leptomeninges. Primary suprasellar melanocytomas are exceedingly rare, with only a handful of cases reported. The systemic spread of a nontransformed meningeal melanocytoma is an unusual occurrence. Herein, we report the first case of a primary sellar melanocytoma with cerebral and spinal meningeal seeding. *Case Report*. A 30-year-old male with no previous medical history presented to the endocrinology department with a loss of body hair. The endocrine workup concluded with isolated hypogonadotropic hypogonadism. The Magnetic Resonance Imaging (MRI) of the brain and sella revealed a large suprasellar mass continuous with the infundibulum of the pituitary gland. It was heterogeneously hyperintense on T1-, T2-, and FLAIR-weighted images and was enhanced with contrast, along with cerebral and spinal leptomeningeal spread. The patient was referred to the neurosurgery department, and a lumbar spine biopsy was indicated. The histopathological examination was suggestive of a grade I meningeal pigmented melanocytoma.

**Conclusion:**

Thus, primary sellar melanocytomas with leptomeningeal spread are an extremely rare phenomenon. Metastatic malignant melanoma should be ruled out. Being aware of differential diagnosis and the unusual behavior of meningeal melanocytoma will be necessary to manage the patient appropriately. Complete tumor resection is the best treatment whenever possible, and radiotherapy should be considered in case of unresectability or partial resection.

## 1. Introduction

Cerebral primary melanocytic tumors are very rare neuroectodermal neoplasms arising from the leptomeninges. According to the World Health Organization (WHO) in 2007, primary melanocytic neoplasms present 4 distinct pathological entities that can manifest diffusely as leptomeningeal melanosis and melanomatosis or focally as meningeal melanocytoma, and primary melanoma [1]. Among these, melanocytomas are very uncommon [2, 3]. Primary meningeal melanocytomas of the central nervous system (CNS) which were first described by Park et al. [4] have an estimated incidence of 1 per 10 [5]. Primary suprasellar melanocytomas are exceedingly rare, with only a handful of cases reported [6, 7].

The systemic spread of a nontransformed MM is an exquisitely rare occurrence [8, 9].

Herein, we report the first case of a primary suprasellar melanocytoma with cerebral and spinal leptomeningeal seeding with the longest follow-up in the literature of 9 years without surgical resection or radiotherapy.

## 2. Case Report

A 30-year-old male with no previous medical history presented to the endocrinology department in 2013 with loss of body hair. The clinical examination showed a loss of axillary and facial hair (beard). The general examination of the patient was unremarkable. In particular, the neurological exam was normal.

The endocrine workup showed decreased testosterone levels with inappropriate gonadotropin levels (testosterone: 0.18 ng/mL [3-12 ng/mL], FSH: 0.63 mUI/mL [1.3-11 mUI/mL], and LH: 0.59 mUI/mL [1.1-10 mUI/mL]) and a slightly elevated prolactin level (20.3 ng/mL [2-10 ng/mL]) concluding to an isolated hypogonadotropic hypogonadism (TSH: 0.99 *μ*UI/mL [0.25-4.5 *μ*UI/mL] and FT4: 16.11 pmol/L [9-24 pmol/L]). The 1 *μ*g synacthen test revealed appropriate cortisol levels in response to the stimulation test.

**Figure 1:**
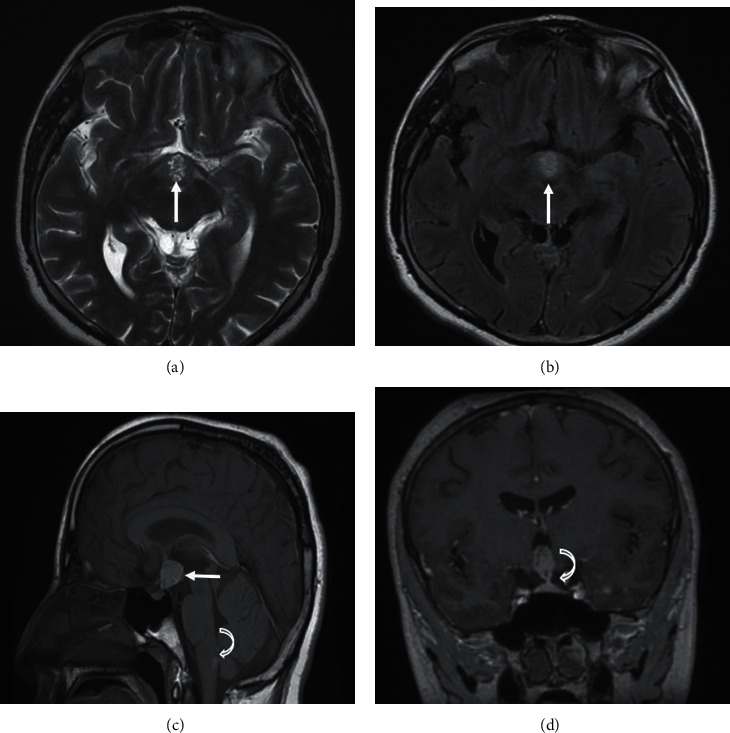
Multisequential multiplanar magnetic resonance images of the brain with and without contrast: axial T2 (a), axial FLAIR-weighted images (b), sagittal T1 (c), and contrasted coronal T1 (d). MRI images reveal a large mass centered on the suprasellar mass, continuous with the infundibulum of the pituitary gland. It is heterogeneously hyperintense on T1-, T2-, and FLAIR-weighted images (arrows in (a–c)), slightly enhanced. Normal pituitary tissue is seen in the bass of the fossa (curved arrow (d)) and a subarachnoid nodular mass in the medulla oblongata (curved arrow (c)).

**Figure 2:**
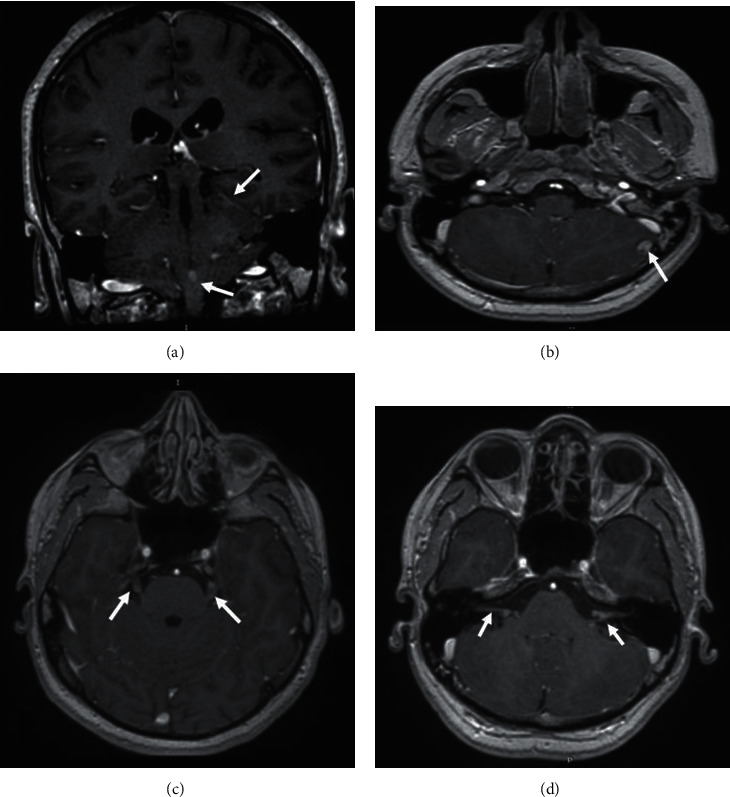
Multisequential multiplanar magnetic resonance images of the brain with contrast. Coronal (a) and axial (b–d) contrasted T1 images reveal multiple subarachnoid nodules slightly enhanced (a, b), bilateral nodular lesions in the trigeminal nerves (c), and acoustic-facial bundles (d).

**Figure 3:**
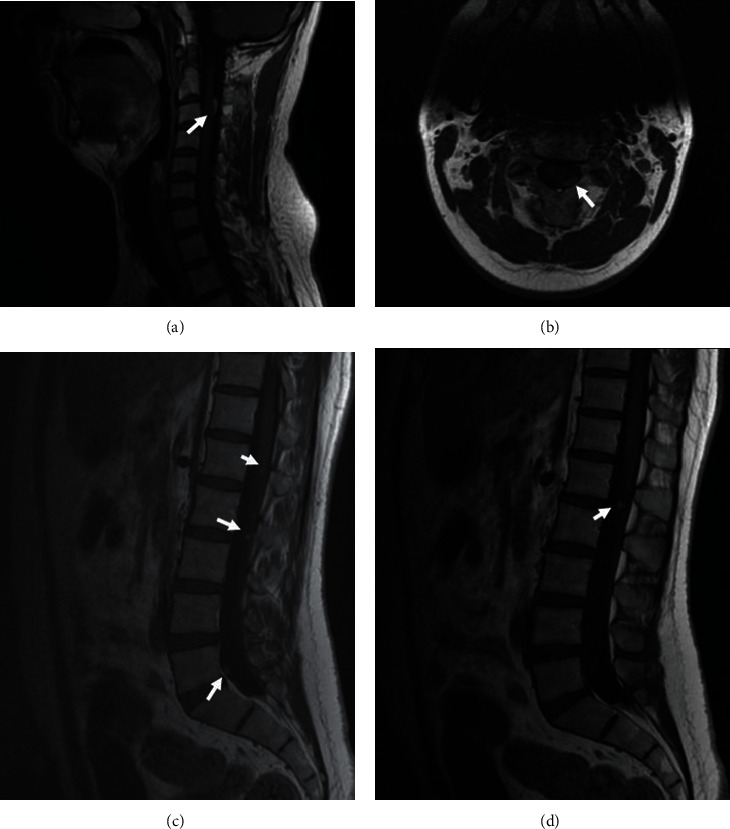
Multisequential multiplanar magnetic resonance images of the spinal cord without contrast. Sagittal (a) and axial (b) T1-weighted MR images reveal an intradural extramedullar mass having a homogeneous high signal intensity, which is consistent with the T1-shortening effect of melanin located at the level of C3 (arrow). Sagittal T1-weighted MR images (c, d) reveal multiple intradural extramedullar small nodules with high signal T1 intensity (arrow) in the cauda equina.

The ophthalmologic examination showed preserved visual acuity and a normal fundus examination. MRI of the brain and sella showed a large suprasellar mass with cerebral and spinal leptomeningeal spread (Figures 1–3).

A lumbar puncture revealed no pathological findings. Polymerase Chain Reaction (PCR) evaluation for Mycobacterium tuberculosis in the cerebrospinal fluid (CSF) was negative. Tumor markers in the blood and the CSF were also negative. The converting enzyme dosage revealed normal levels, making the diagnosis of sarcoidosis unlikely. A thoracoabdominal-pelvic CT scan did not show any associated lesions.

The patient was referred to the neurosurgery department, and a biopsy of the arachnoid leaflet and the dura mater was taken. On exploration, the arachnoid was thick and blackish.

The histopathological examination was suggestive of a grade I meningeal pigmented melanocytoma.

An androgen replacement therapy was instaured, and then, a dopaminergic agonist (bromocriptine) was added due to the increase in prolactin levels reaching 50-60 ng/mL.

Total resection of the tumor, as well as the meningeal spread, was not possible. The patient refused any radiotherapy. Owing to the benign nature of the lesion and the absence of neurological or ophthalmological impairment, we opted for clinical and radiological monitoring. The patient was then lost to follow-up.

**Figure 4:**
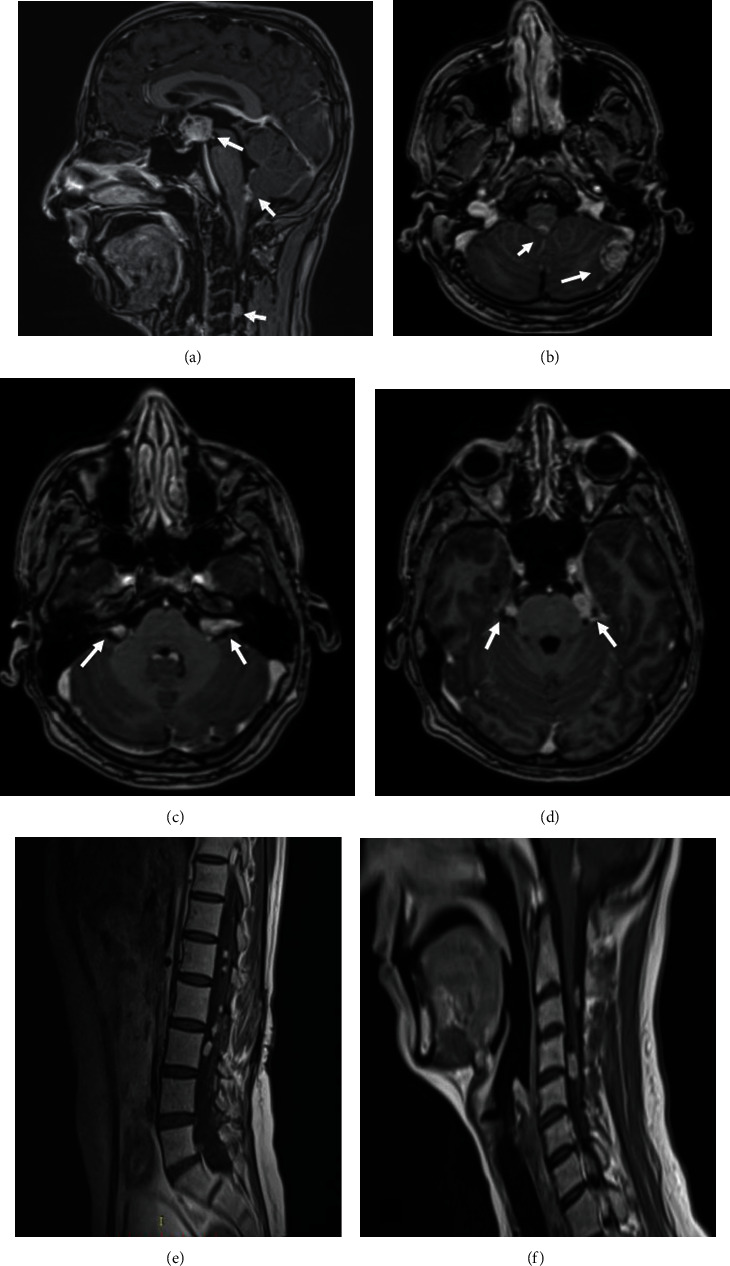
Multisequential multiplanar magnetic resonance images of the brain and the spine with and without contrast. Sagittal (a) and axial (b–d) contrasted T1 brain images and sagittal cervical and lumbar spine (e, f) T1 images reveal an increase in the size of the suprasellar tumor (a) and an increase in the number and size of the meningeal nodular masses in the brain (b–d) and the spine (e, f).

**Figure 5:**
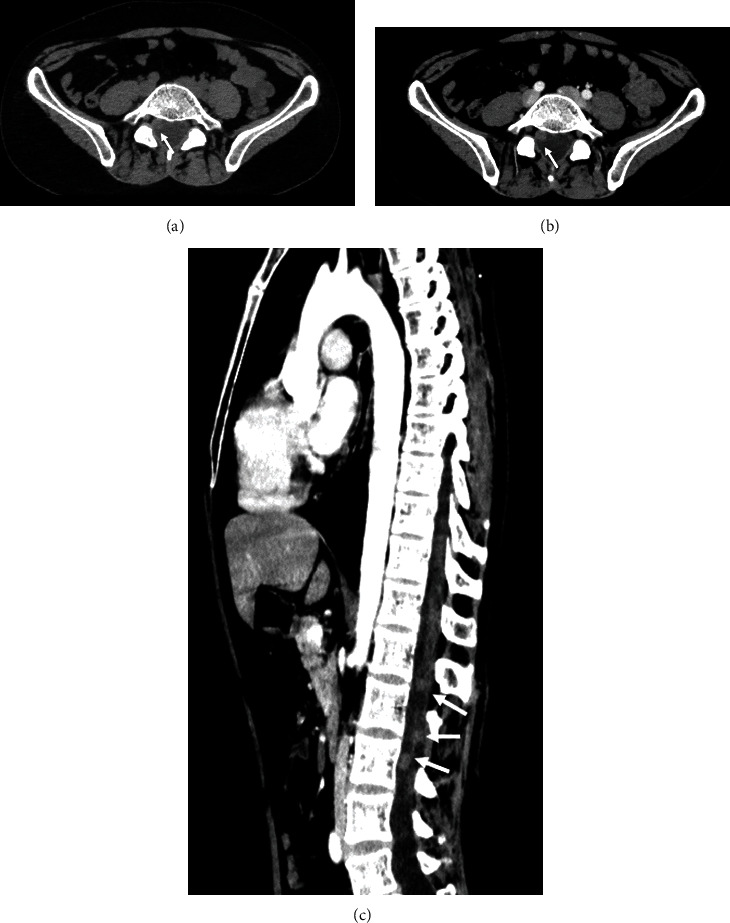
Thoracic and abdominal CT scan with and without contrast. Unenhanced axial CT scan image (a) shows a well-defined hyperdense intraspinal mass (arrow). Axial (b) and sagittal (c) contrast-enhanced CT images show homogeneously enhanced multiple intraspinal masses (arrows).

He consulted in 2020 for a sudden drop in visual acuity. The fundoscopy showed stage 3 papillary edema. The neurological exam of the patient was normal. An MRI was urgently done to reveal an increase in the size and number of leptomeningeal seeding complicated by obstructive hydrocephalus (Figure 4). The CT scan showed no abnormalities other than well-defined intraspinal masses (Figure 5). The patient was urgently rereferred to neurosurgery, and treatment with radiotherapy is currently being discussed.

## 3. Discussion

According to the World Health Organization (WHO) in 2007, primary melanocytic neoplasms present 4 distinct pathological entities that can manifest diffusely as leptomeningeal melanosis and melanomatosis or focally as meningeal melanocytoma, and primary melanoma. They are thought to arise from leptomeningeal melanocytes [1].

The medulla oblongata and the upper cervical levels present the highest concentration of melanocytes. Thus, these tumors are mostly encountered in the spinal column and infratentorial compartment [3, 5]. In cases with an intraparenchymal localization, the melanocytes most probably arise from the Virchow-Robin space [10].

Primary meningeal melanocytomas of the CNS have an estimated incidence of 1 per 10 million with a slight female predominance [5].

MM outside the spectrum of cutaneous melanosis, as seen in our patient, is commonly diagnosed due to its compressive effect on the surrounding nervous structures [11].

Our patient had no neurological symptoms at the time of diagnosis but then developed signs related to hydrocephalus.

The sellar melanocytoma is exceedingly rare, clinically mimicking other sellar masses such as adenoma, craniopharyngioma, germinoma, or metastasis. Different clinical features were reported, such as headaches, visual field, ophthalmoplegia and ptosis, hypopituitarism, and diabetes insipidus [12].

Isolated hypogonadotropic hypogonadism revealing a suprasellar melanocytoma, as shown in this case, is an extremely rare occurrence. Only a handful of cases have been reported [6, 13]. The gonadotropic deficit was due to hypothalamic involvement. The elevated level of prolactin was attributed to “the stalk effect”; a compressed pituitary stalk causes a decrease in dopamine release that subsequently leads to unopposed prolactin secretion.

Due to the rarity of primary sellar melanocytic tumors, most cases were preoperatively misinterpreted as nonsecretory, possibly hemorrhagic, pituitary adenomas [13].

The typical macroscopic appearance of MM is a well-encapsulated, dark lesion, heavily pigmented, and firmly attached to the leptomeninges. Ultrastructurally, MM contains a large number of melanosomes and premelanosomes at different stages of differentiation [14].

Liubinas et al. [2] classified melanocytic lesions of the CNS into high-grade melanomas and low-grade melanocytomas. Only a few cases in their study exhibited moderately increased cellularity and mitotic activity. Thus, they were classified as intermediate-grade lesions. According to Brat et al., these groups represent different histological entities. The transformation from a low-grade or intermediate-grade to a high-grade lesion is impossible. However, this theory has been denied by Roser et al. who reported a case of MM degenerating into malignant melanoma 12 years later [15] along with two other cases of malignant transformation of spinal melanocytomas which have been reported earlier in the literature.

The imaging appearance of MM is variable depending on the concentration of melanin. At CT, MM can manifest as an extra-axial iso- to hyperattenuating lesion and enhances homogeneously after administration of contrast material. This appearance can mimic meningiomas. However, unlike meningioma, MM has no calcification or hyperostosis of the adjacent bone.

On MRI, the tumor exhibits homogeneous high intensity on the T1-weighted image and low intensity on the T2-weighted image. This signal pattern is unique and opposite to the usual signal of intracranial tumors due to the paramagnetic effect of the high melanin content [16]. These tumors usually enhance homogeneously after contrast administration. Although the enhancement of the mass may not be obvious to the eyes, as the mass is hyperintense on T1-weighted imaging, homogenous enhancement may be confirmed by subtraction imaging. The lack of high signal on T1-weighted images can be the result of the cellular or fibrous nature of the tumor [17].

Unfortunately, it is sometimes hard to distinguish melanocytomas from other extra-axial tumors, such as meningiomas, schwannomas, and malignant melanoma.

Leptomeningeal seeding is a rare phenomenon that has been shown to occur late in the course of MM as a result of malignant transformation [15] or a distinct aggressive course without malignant pathological features [18].

The histopathological examination of the spinal lesion, in our case, did not show any anaplastic features or mitosis, which virtually eliminates the possibility of malignant transformation.

Despite the benign nature of MM, its management is complicated due to the high risk of metastases and local invasion. Complete tumor resection is the best treatment in terms of both local control and survival. When it cannot be achieved, postoperative radiotherapy should be strongly considered.

## 4. Conclusion

Thus, primary sellar melanocytomas with leptomeningeal spread are an extremely rare phenomenon. Metastatic malignant melanoma should be ruled out.

Being aware of differential diagnosis and the unusual behavior of meningeal melanocytoma will be necessary to manage the patient appropriately.

Complete tumor resection is the best treatment whenever possible, and radiotherapy should be considered in case of unresectability or partial resection.

## Data Availability

The data used to support the findings of this study are included within the article.
